# Floral display and habitat fragmentation: Effects on the reproductive success of the threatened mass‐flowering *Conospermum undulatum* (Proteaceae)

**DOI:** 10.1002/ece3.5653

**Published:** 2019-09-26

**Authors:** Nicola Delnevo, Eddie J. van Etten, Margaret Byrne, William D. Stock

**Affiliations:** ^1^ Centre for Ecosystem Management Edith Cowan University Joondalup WA Australia; ^2^ Biodiversity and Conservation Science Department of Biodiversity, Conservation and Attractions Bentley Delivery Centre Bentley WA Australia

**Keywords:** biodiversity hotspot, conservation, floral display, germination, isolation, mating system, population size, seed set

## Abstract

1. Fragmentation of natural vegetation is currently one of the largest threats to plant populations and their interactions with pollinators. Plant reproductive susceptibility to habitat fragmentation has been investigated in many species; however, the response of wild mass‐flowering species is poorly known, with research limited to mainly boreal plant species.
2. Here, we studied twelve remnant populations of the threatened mass‐flowering shrub *Conospermum undulatum* in the southwest Australian biodiversity hotspot, each presenting different population size, level of isolation, and floral display. We assessed the impact of fragmentation on (a) fruit and seed production; and (b) seed germination. To gain a deeper understanding of factors influencing the reproductive success of *C. undulatum*, we performed pollinator exclusion and self‐pollination treatments to experimentally assess the mating system of this threatened shrub.
3. We found *C. undulatum* to be strictly self‐incompatible and totally reliant on pollinators visiting with an outcrossed pollen load to complete the reproductive cycle. Further, we found that fruit production dropped from 35% to <20% as a result of decreasing floral display. A reduction in population size from 880 to 5 plants and from ~700 to 0.21 in the floral display index led to a decrease in seed output, while a similar reduction in seed output, from 6% to 3%, was observed as a result of increasing isolation index from −21.41 to −0.04. Overall, seed germination was positively related to population size, and a negative relationship was found between germination and isolation.
4. *Synthesis and applications*. Our results demonstrate the important relationship between pollinators and floral morphology in plants of southwest Australia that have coevolved with native pollinators and developed characteristic flower morphologies over long time frames. Indeed, due to its characteristic pollination mechanism, the self‐incompatible *C. undulatum* can only rely on specialized native pollinators for pollen flow and cannot rely on its mass‐flowering trait to attract generalist pollinators from coflowering species; neither can it compensate for the lack of visitors by promoting geitonogamy. Consequently, fragmentation has a significant effect on the reproductive output of *C. undulatum*, and size, isolation, and floral display of populations are important factors to be considered when planning conservation actions for the species.

## Graphical abstract

Plant reproductive susceptibility to habitat fragmentation has been investigated in many species; however, the response of wild mass‐flowering species is poorly known. Here, we studied the reproductive output and mating system of twelve remnant populations of the threatened mass‐flowering shrub *Conospermum undulatum* in the southwest Australian biodiversity hotspot, each presenting different population size, level of isolation, and floral display. Fragmentation has a significant effect on the reproductive output of *C. undulatum*, and size, isolation, and floral display of populations are important factors to be considered when planning conservation actions.

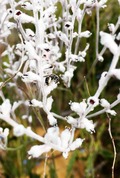

## 1 INTRODUCTION

Pollinators visiting flowers with adequate amounts of pollen grains are an essential requirement for pollen dispersal and, ultimately, reproduction for ca. 87% of the world's flowering plant species (Ollerton, Winfree, & Tarrant, 2011; Winfree, Bartomeus, & Cariveau, 2011). Flowering plants are a crucial component of most terrestrial ecosystems (Ollerton, Johnson, & Hingston, 2006), and the rich biodiversity of such systems relies on these plants and their interactions with pollinators. It is widely known that reproduction by seeds has a key role for fitness, migration, adaptation, and ultimately population persistence of plant species (Fenner & Thompson, 2005). Yet, as a consequence of global change, many plant and pollinator populations are declining (Biesmeijer et al., 2006), with mutualistic plant–pollinator interactions frequently disrupted (Thomann, Imbert, Devaux, & Cheptou, 2013), which can have direct effects on plant population viability. Fragmentation of vegetation is one of the most pervasive changes in terrestrial ecosystems that affects plants and their pollinators. The rate at which natural habitats have been fragmented by clearing for urban and agricultural land uses has increased substantially during the last 60 years and now is at unprecedented levels (Ellis, Goldewijk, Siebert, Lightman, & Ramankutty, 2010). Based on a meta‐analysis of plant reproductive susceptibility to habitat fragmentation, Aguilar, Ashworth, Galetto, and Aizen (2006) suggested that a decrease in size and connectivity of plant populations resulting from habitat fragmentation could locally reduce the reproductive success of plants. Indeed, small and/or isolated fragments of plant populations may be less attractive for pollinators (Dauber et al., 2010; Delmas, Escaravage, & Pornon, 2014), leading to a reduction in both pollen *quantity* (i.e., decrease in pollination events) and pollen *quality* (i.e., less deposition of conspecific and outcrossed pollen grains on stigmas; Aizen & Harder, 2007; Eckert et al., 2010). Pollen quality is particularly important for self‐incompatible species that lack the reproductive assurance that self‐reproduction may provide (Morgan & Wilson, 2005). In addition, a factor that has rarely been considered, especially for conservation purposes, is how plant species that rely on massive population floral display for attracting pollinators (i.e., mass‐flowering species; Heinrich & Raven, 1972) respond to habitat fragmentation.

*Conospermum* (Proteaceae) is an endemic genus to Australia with its center of distribution being southwest Western Australia (Bennett, 1995). The southwest Australian Floristic Region (SWAFR; Hopper & Gioia, 2004) encompasses an exceptional concentration of endemic flora and is recognized as a global biodiversity hotspot (Mittermeier et al., 2004; Myers, Mittermeier, Mittermeier, da Fonseca, & Kent, 2000) and has been impacted by fragmentation because of urban and agricultural development. *Conospermum undulatum* is a mass‐flowering species, and during the reproductive season, its white inflorescences dominate the (nonfragmented) landscape resembling drifting smoke; hence, its common name is smoke bush. This species is currently listed in the threatened flora of Western Australia (W.A Government Gazette, 2018) and has been assessed as “Vulnerable” using IUCN red list criteria (Department of Environment & Conservation, 2009).

In general, mass‐flowering crops and native species have been shown to be attractive to a larger diversity of pollinators and may attract a higher abundance of floral visitors from surrounding flowers (Hegland & Totland, 2005; Westphal, Steffan‐Dewenter, & Tscharntke, 2003). Therefore, it may be expected that mass‐flowering plants may not be impacted by the detrimental effects of fragmentation by remaining highly attractive to a large pool of pollinators due to their high flower abundance. However, this hypothesis has only been tested on crops and boreal plant species (e.g., Diekötter, Kadoya, Peter, Wolters, & Jauker, 2010; Mitchell, Karron, Holmquist, & Bell, 2004), that are pollinated by honeybees (*Apis mellifera*) and bumble bees (*Bombus* sp.), important pollinators in Europe. Results from these studies may not be transferable to plants in the SWAFR where plant–pollinator interactions have evolved over long time frames. Plants within the SWAFR have coevolved with different pollen vectors such as birds, mammals, and small native bees, leading to the development of specific flower morphologies and pollination systems. *Conospermum undulatum* plants possess small and characteristic flowers with an active pollination mechanism described by Holm (1978) that involves a tactile stimulation within the calyx tube to trigger the stigma, so it makes contact with the visitor. Houston (1989) reported the identification of a southwestern Australian species group of bees (*Leioproctus conospermi*), which consists of three species oligolectic on flowers of *Conospermum* that possess morphological adaptations to enable this remarkable pollination. He also reported that, besides these native bees, smoke bush flowers are visited by argid sawflies (Argidae), and flies of families Bombyliidae and Syrphidae, although the true pollinators remain uncertain. The majority of other common generalist pollinators, such as Dipterans, are unable to produce an effective pollination (i.e., untriggered style and nondehisced anthers, or insect trapped fatally by the triggered style). Further, the most abundant insect pollinator in the SWAFR, the introduced European honeybee (Phillips, Hopper, & Dixon, 2010), is too large to pollinate the flowers of the smoke bush (N. Delnevo, personal observation).

In addition, within small populations of mass‐flowering species, pollinators tend to have higher numbers of within‐plant floral visits compared with those in larger populations (Eckert, 2000), with a consequent increase in levels of geitonogamy (i.e., transfer of pollen between different flowers of the same plant). Such transfer of self pollen may represent a reproductive assurance to compensate for the lack of outcross pollen, but this would depend on the strength of inbreeding depression (Campbell & Husband, 2007). However, many genera of Proteaceae exhibit self‐incompatibility systems and evidence of selective fruit development (Goldingay & Carthew, 1998; Vaughton & Carthew, 1993). Accordingly, *C. undulatum* is considered to be a self‐incompatible species (Goldingay & Carthew, 1998; Morrison, McDonald, Bankoff, & Quirico, 1994), and this would reduce the reproductive assurance of geitonogamy, especially in small fragments. However, the reproductive biology of this species has not been studied in detail as yet, and there is a need to understand the reproductive responses of this rare plant in a highly fragmented landscape to inform future conservation efforts. Here, we studied the effects of fragmentation on the reproductive biology of *C. undulatum* to inform conservation. Specifically, we asked (a) were fruit production and seed production related to aspects of fragmentation?; (b) was seed germination following the same trends?; and (c) to what extent was geitonogamy evident in the mating system of *C. undulatum*? We expected small populations in isolated fragments with low floral display to produce fewer fruits and seeds compared with large, connected, highly visible populations due to a lack of pollen quantity and quality. Consequently, if pollen‐mediated gene flow is not able to extend the mating pool beyond the single fragment providing genetic rescue in small and isolated population from the effects of inbreeding, then the number of germinants should also be related to our population descriptors. Finally, as for many proteaceous species, we expected our target species *C. undulatum* to be self‐incompatible and therefore reproductive assurance via autogamy would be zero to inconsequential. However, even if the trigger mechanism of the stigma is a physical barrier to self‐pollination, geitonogamy may still occur, especially in small populations, making predictions less clear.

## 2 MATERIALS AND METHODS

### 2.1 Study site and species

**Figure 1:**
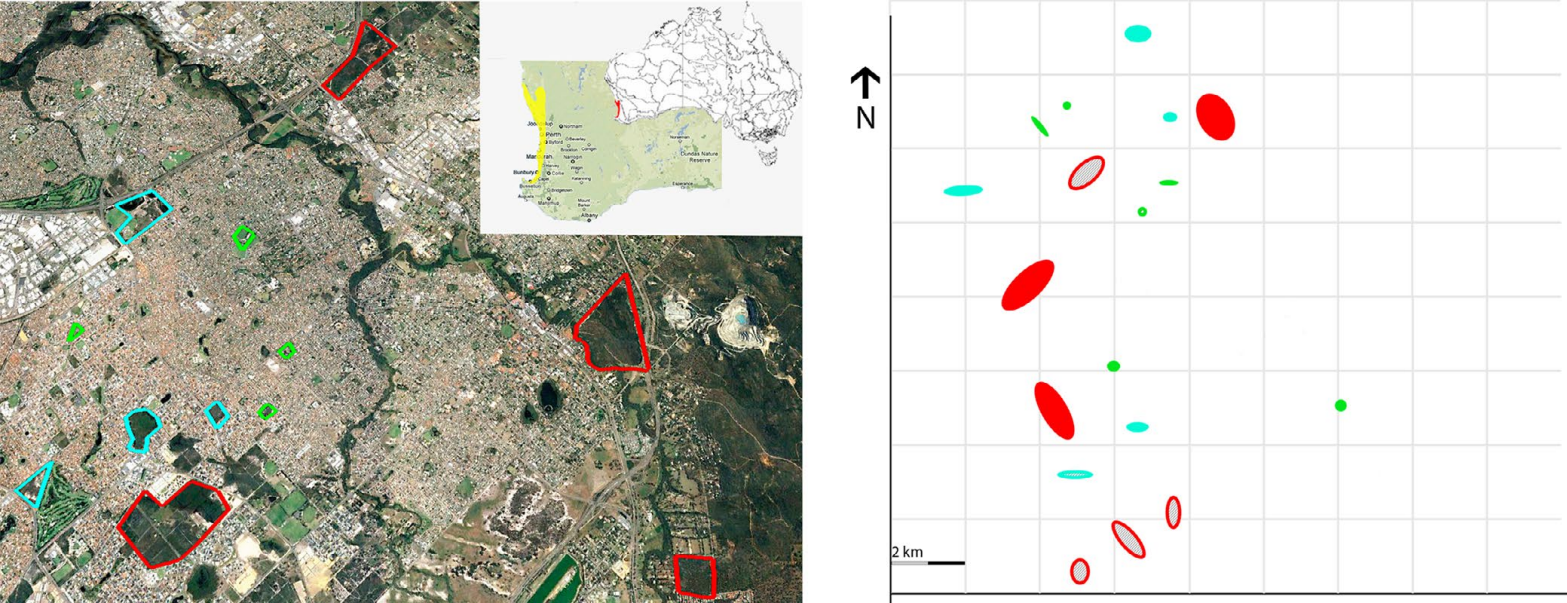
Left: example of fragmented bushland in an urban matrix in the Swan Coastal Plain. Right: spatial disposition of all extant *Conospermum undulatum* populations. Filled circles are populations selected for this study, and empty circles are population not selected; large remnants are highlighted in red, medium‐sized in light blue, and small in green. A precise map cannot be provided for Threatened flora

The study was conducted in southwest Western Australia within the Swan Coastal Plain bioregion (Figure 1). This region is a low lying coastal plain that extend from Jurien Bay, north of Perth, to Cape Naturaliste in the south, and it is part of the southwest Australia biodiversity hotspot (Mittermeier et al., 2004). The Swan Coastal Plain was historically cleared for agriculture and forestry, and is now experiencing extensive land clearing for urbanization. Urbanization has more than doubled since the 1970s, is centered around Perth, the capital city of Western Australia, and has impacted biodiversity of the region (e.g., Davis, Gole, & Roberts, 2013). Urban expansion has reduced natural or seminatural vegetation on the Swan Coastal Plain to 34.7%, with only 10% in protected areas (Wardell‐Johnson et al., 2016). Our target species, *C. undulatum*, is a threatened, naturally rare plant species with a range restricted to ca. 55 km^2^ in an expanding urban zone.

*Conospermum undulatum* is a monoecious plant which grows as an erect, compact shrub up to 1.5 m tall with distinctive fibrous, longitudinally fissured stems and glabrous leaves to 12 cm long and 3.8 cm wide; leaves have characteristic undulating margins. It was originally considered to be a variety of *Conospermum triplinervium*, which also occurs in the region but with different habit (i.e., *C. undulatum* never develops a thick trunk and is typically multi‐stemmed) and leaf morphology (Bennett, 1995). Molecular evidence has established *C. undulatum* as a distinct species (Close et al., 2006) and recently developed genetic resources will further clarify its genetic relationships (Delnevo, Piotti, van Etten, Stock, & Byrne, 2019). Our target species is classified as resprouter; hence, it can survive fire by regenerating from rootstock. The hermaphroditic woolly flowers have long, white hairs and are produced in inflorescences held well above the leaves. The flowering period usually ranges from late August to late October. Fruits are cone‐shaped, covered with tan orange hairs and contain only one seed (Bennett, 1995). *Conospermum undulatum* is an entomophilous species and possesses an active pollination mechanism that involves a tactile stimulation within the calyx, which causes the style to flick down on the back of the insect, and simultaneously, causing the fertile anthers to dehisce explosively, casting pollen onto the visitor (Holm, 1978; see Douglas (1997) for a morphological description). Thus, its flowers need to be visited by insects carrying a suitable pollen load for pollination to occur leading to develop fruits.

### 2.2 Data collection

Prior the beginning of the flowering season, in August 2017, we recorded the GPS location of every individual plant in all the existing populations of *C. undulatum*. Then, by means of ArcGIS (ESRI, Redlands, USA) we characterized each population by their size (i.e., number of *C. undulatum* plants), fragment area, and percentage of vegetated (native and not native) land within a 500‐m‐radius area around the population centroid. Since the foraging range of bees is related to body size (Greenleaf, Williams, Winfree, & Kremen, 2007), a radius of 500 m was selected based on the fact that small native bees, with restricted ranges, were the most likely pollinators of our target species. Subsequently, we calculated an isolation index based on a modified version of the incidence function model (Hanski, 1994). This model accounts both for distances to all possible neighboring populations, and the area of those populations, providing a better estimate in highly fragmented habitats and small datasets compared with either nearest neighbor or buffer measures (Moilanen & Nieminen, 2002). Population isolation was calculated as: $S_{i}=-\sum_{j\neq i}\exp\left(-\alpha d_{\mathrm{ij}}\right)\cdot A_{j}^{b}$, where *S_i_* is the isolation of the patch *i*; *α* is a scaling parameter for the effect of distance to migration (1/*α* is the average migration distance); *d_ij_* represent the distance between fragment *i* and *j*; *A_j_* is the area of fragment *j*; b is a scaling parameter of immigration as a function of the area of fragment *j*. Again, since *C. undulatum* seeds are gravity dispersed (Close et al., 2006) and small native bees are the likely main pollen vector, we estimated an average migration distance up to 500 m (Campbell & Husband, 2007). However, isolation may be both spatial and temporal. Indeed, flowering time is highly relevant as it is the first mechanism of reproductive isolation. To account for possible effects of temporal isolation, we recorded the reproductive phenology of this species once a week for the entire flowering season and we evaluated the flowering synchrony between populations using a modified version of the method proposed by Freitas and Bolmgren (2008), replacing individuals with populations. Overall, populations were synchronous with a score of 0.53 on a scale from 0 to 1, being 0 asynchrony, 0.25 low synchrony, 0.5 synchrony, and 1 perfect synchrony (Figure S1). Thus, there was no temporal isolation between populations. Finally, during the flowering season we counted the total number of inflorescences of each individual in populations with less than 20 plants, and from 20–40 randomly selected individuals in larger populations. We then estimated the floral display of each *C. undulatum* population as: FD = (*I* • *A*_C_)/100, where FD is the floral display index of a population; *I* is the mean number of inflorescences per plant in the specific population; and *A*_C_ is the area (in m^2^) covered by *C. undulatum* plants within the fragment (obtained through ArcGIS using the minimum convex polygon method). *Conospermum undulatum* seeds are gravity dispersed, and plants appear in clumps of similar density across all populations. Therefore, due to the biology of the species, plant density was not informative and was not considered further in this study.

From a total of 18 remnant populations of *C. undulatum*, we selected 12 populations encompassing the entire range of population sizes and levels of isolation. Since *C. undulatum* is a threatened species, license conditions restricted collections to 20% of fruits per plant from 20% of plants in a population. So, at the end of the flowering season when flowers began to senesce, we collected fruits (and seeds) from randomly placed bags around five inflorescences per plant in 20–40 randomly selected plants per population. In small populations with <20 plants, we bagged all the individuals; however, only seeds from 20% of the plants were kept, the rest was returned to the population of origin after being recorded. In the laboratory, we counted the number of flowers, fruits, and seeds collected for each plant (total of 65,020 flowers and 2,505 seeds from 210 selected plants). The number of flowers was assessed by counting the scars left on the white, woolly inflorescence stalk.

Seed viability was assessed by carefully nicking off a small portion of the fruit wall under a dissecting microscope. Seeds with firm, white embryo were classified as viable, as opposed to seeds with rotting embryos. Also, nicking the fruit wall is part of the recommended method for germinating *C. undulatum* seeds (Cochrane, 2007). Nicked viable seeds were placed in a 10% plant preservative mix (Plant Cell Technology) for 10 min to prevent the formation of mold on the exposed embryo. Then, following the best‐known germination treatment (A. Crawford, personal communication, 2017) we soaked the seeds in 10% Regen2000© smokewater (Grayson Australia) for 24 hr before sowing them on 75% agar with 100 mg/L of gibberellic acid solution, to aid germination. Seeds were placed in a germination chamber with 12 hr of daily photoperiod at 15°C and scored for radicle emergence every 2 weeks for 9 months. All seeds from each mother plant were kept separate.

To experimentally assess the extent of self‐compatibility in *C. undulatum*, we performed three experimental treatments in the field: pollinator exclusion (PE), pollinator‐excluded triggered flowers without pollen supplementation (PET), and hand self‐pollination on pollinator‐excluded flowers (PES). In a medium‐sized population of *C. undulatum* (216 plants), we randomly selected ten plants per treatment 2 weeks before anthesis, and we placed fine mesh bags around three inflorescences per plant. In this way, we prevented insects from visiting the flowers (i.e., PE treatment). During anthesis, we triggered the stigma of PET flowers, and we hand‐pollinated flowers of PES treatment with pollen from different flowers on the same plant by means of a 1‐mm flathead screwdriver as this enabled us to reach the stigma.

### 2.3 Data analysis

Following data exploration, we removed fragment area and percentage of vegetated land around the population centroid from the variables list because of high multicollinearity, with a variance inflation factor (VIF) of 44.35 and 31.43, respectively. The variables retained were population size, isolation, and floral display which had no collinearity, with a VIF below the selected cutoff value of 2.5 (Zuur, Ieno, Walker, Saveliev, & Smith, 2009). Separate GLMs were fitted for the following response variables: (a) proportion of fruit production; (b) proportion of seed production; and (c) proportion of germinated seeds. To account for non‐normal distribution of residuals, nonhomogeneous variances, and moderate overdispersion, we used quasi‐binomial error distributions (appropriate for proportional data) and checked that the assumptions were fulfilled by visual inspection of residual patterns (Zuur et al., 2009). Full models for fruit and seed production contained all the retained population descriptors (i.e., population size, isolation, and floral display) as the explanatory variables. Some small populations produced no viable seeds to be tested for germination, and so, we removed those populations from the dataset of the third model (i.e., proportion of germinated seeds) because this could not be determined. However, by doing so, floral display presented a collinearity issue, with VIF above the 2.5 cutoff value; thus, we removed this variable from the relative full model. Starting from each of the three full models, model selection was then performed by excluding nonsignificant terms. Furthermore, the absolute value of the standardized regression coefficient (ß) of each scaled explanatory variable can be a useful metric for determining the relative importance of the respective predictors (Murray & Conner, 2009). Each explanatory variable was scaled by subtracting its mean and dividing by its standard deviation. We used separated models to rank the predictors. All statistical analyses were performed with R version 3.5.2 (R Development Core Team, 2018).

## 3 RESULTS

### 3.1 Self‐pollination

The results of the self‐compatibility experiments are outlined first as these provide an important basis for understanding the results of the reproductive success analyses. Total insect exclusion treatment (PE) yielded zero fruits (and zero seeds) in all the ten replicates (Table 1). Similarly, even if the stigma was triggered, PET treatment resulted in zero fruits (and zero seeds). Together, these two treatments (PE and PET) demonstrate *C. undulatum* flowers do not self‐pollinate and develop fruit unless visited by insects carrying a suitable load of pollen from previous floral visits. The hand self‐pollination treatment (PES) produced fruits among the ten replicates (Table 1), with an average proportion of success of 0.264 (±0.105). However, all the fruits contained aborted embryos and zero viable seeds developed.

**Table 1:** Reproductive output of *Conospermum undulatum* in term of fruit and seed production for pollinator exclusion (PE), exclusion and triggered flowers (PET), and exclusion and hand self‐pollination (PES) treatments

PE				PET				PES			
Plant ID	Flowers	Fruits	Seeds	Plant ID	Flowers	Fruits	Seeds	Plant ID	Flowers	Fruits	Seeds
1	75	0	0	11	49	0	0	21	10	0	0
2	69	0	0	12	57	0	0	22	10	5	0
3	72	0	0	13	38	0	0	23	18	9	0
4	47	0	0	14	53	0	0	24	45	0	0
5	99	0	0	15	41	0	0	25	40	0	0
6	63	0	0	16	41	0	0	26	10	1	0
7	109	0	0	17	37	0	0	27	28	1	0
8	67	0	0	18	44	0	0	28	10	9	0
9	99	0	0	19	40	0	0	29	10	6	0
10	101	0	0	20	39	0	0	30	14	0	0

### 3.2 Fruit production and seed production

**Figure 2:**
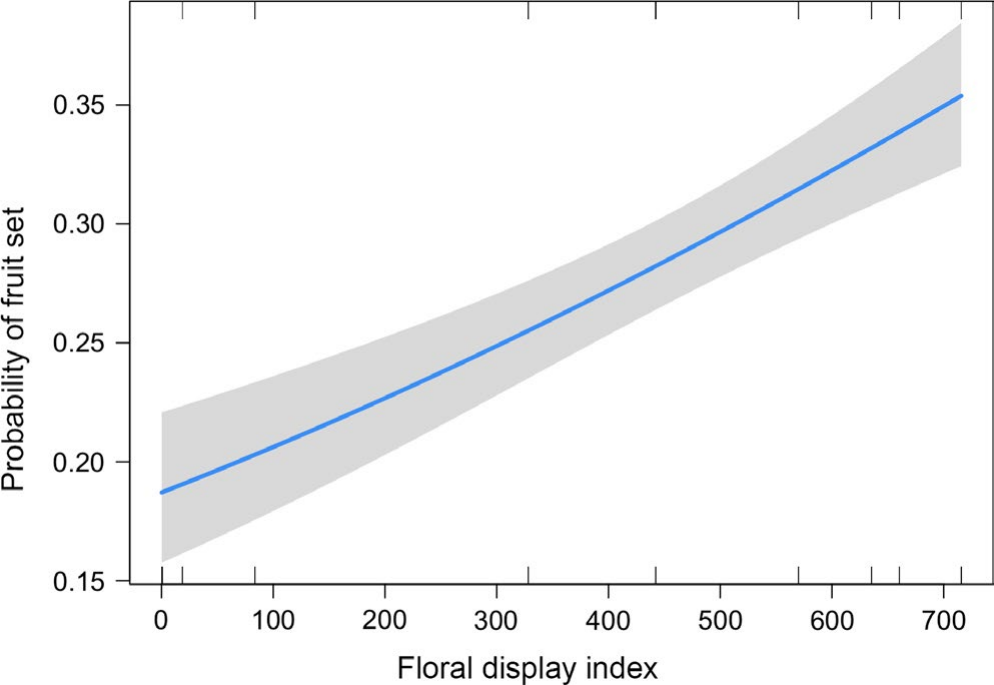
Effect of floral display index on the probability that a flower in *Conospermum undulatum* will develop into a fruit. Confidence intervals are in gray

Population size ranged from 5 to 880 plants (mean = 243.4); fragment area ranged from 0.34 to 51.25 ha (mean = 22.06); isolation index ranged between −0.04 and −21.41 (mean = −7.65); and floral display ranged from 0.21 to 715.70 (mean = 288.02). Fruit production was significantly related to the variability in floral display (*F*_1,175_ = 38.28, *p* < .001), with populations with higher floral display having the largest fruit output, as opposed to less visible populations, where the probability that a flower will develop a fruit dropped by 15 percentage points (Figure 2; Table 2). There was no significant effect of population size (*F*_1,173_ = 0.04, *p* = .835) and level of isolation (*F*_1,174_ = 0.18, *p* = .667); therefore, they were removed from the final model (Table 2).

**Table 2:** Regression parameter estimates for fruit production, seed production, and germination models related to population size, isolation, and floral display variables in *Conospermum undulatum*

	Estimate	Standard error	*p*
Fruit production model			
Intercept	−1.468943	0.105955	<.001***
Floral display	0.001211	0.000201	<.001***
Seed production model			
Intercept	−3.935817	0.1508498	<.001***
Population size	0.0006916	0.0001247	<.001***
Isolation	−0.031324	0.0081266	<.001***
Floral display	0.0007804	0.0001935	<.001***
Germination model			
Intercept	−2.423098	0.218199	<.001***
Population size	0.0009374	0.000281	.001**
Isolation	−0.0415218	0.012637	.001**

*** *p* < .001; **<.01; *<.05; “ .”>.05: Significance codes.

**Figure 3:**
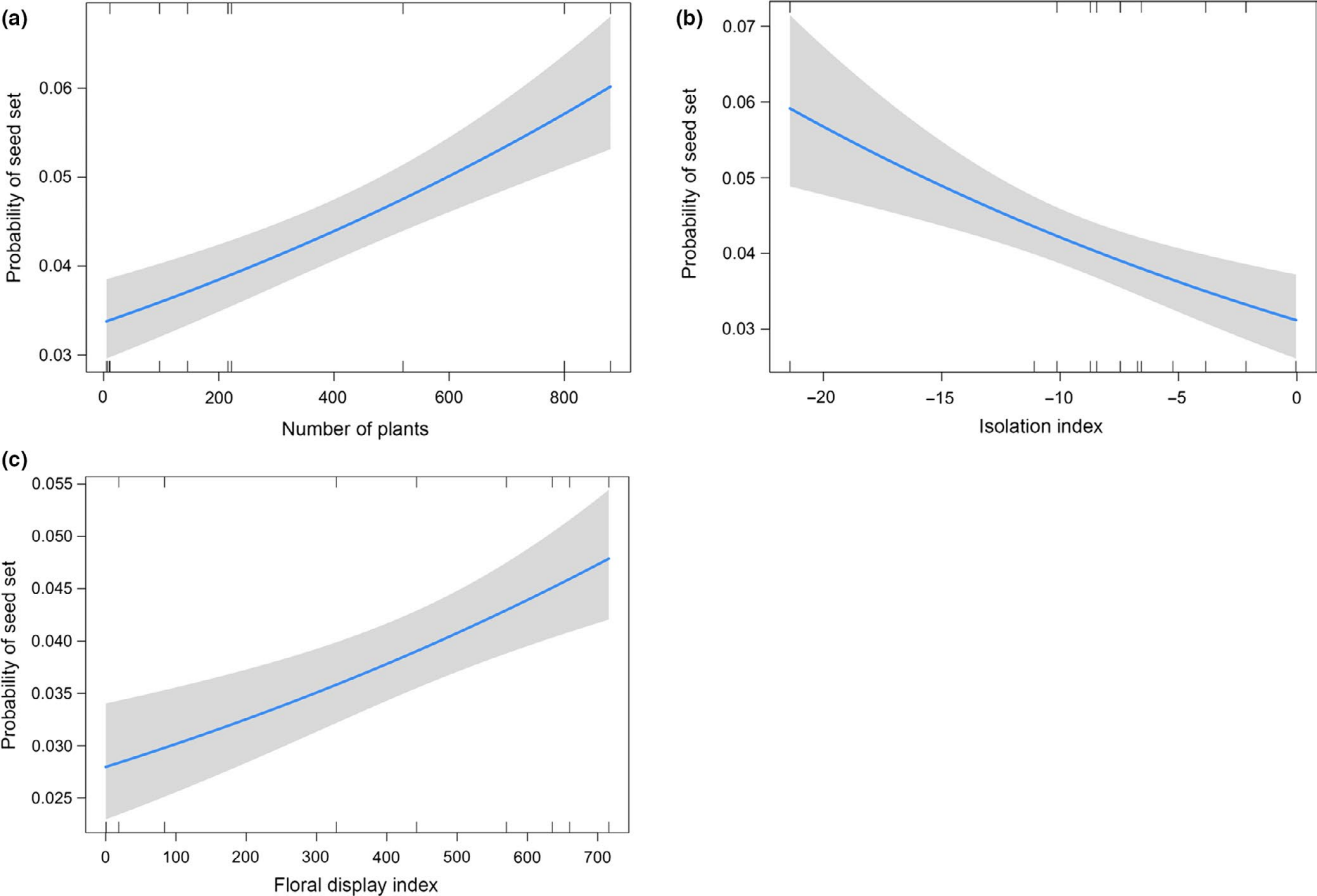
Effect of (a) population size, (b) isolation, and (c) floral display on the probability of a flower in *Conospermum undulatum* to develop a seed. Confidence intervals are in gray

The probability that a flower will develop a seed showed a significant relationship with all the explanatory variables of population size, isolation, and floral display (*F*_1,173_ = 29.80, *p* < .001; *F*_1,173_ = 14.88, *p* < .001; *F*_1,173_ = 16.80, *p* < .001, respectively). In particular, the response variable was positively related to population size (Table 2), with large populations having twice the probability of setting seeds than small populations (Figure 3a). In contrast, the isolation effect was negative (Table 2), but of similar magnitude, with isolated patches having half the probability of setting seeds compared with more connected fragments (Figure 3b). The effect of floral display was positive (Table 2), with the probability that a flower sets a seed increasing from 2.7% to 4.6% between the less visible and more visible populations (Figure 3c). The three explanatory variables population size, isolation, and floral display had a standardized ß coefficient of 0.239, −0.156, and 0.203, respectively (Table S1).

### 3.3 Seed germination

**Figure 4:**
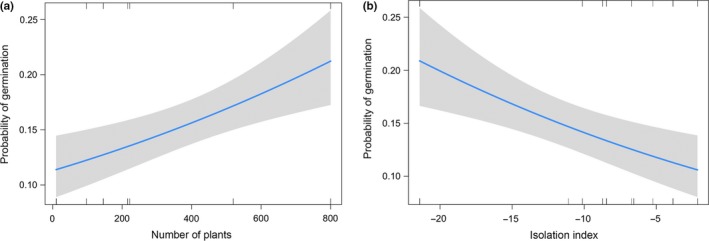
Effect of (a) population size and (b) isolation on the probability of a seed in *Conospermum undulatum* to germinate. Confidence intervals are in gray

*Conospermum undulatum* germination responses are known to be slow and highly variable (A. Crawford, personal communication, 2017). From the 2,505 viable seeds obtained, we recorded 434 radicle emergences (17.33%) in the 9‐month germination period. There were significant effects of population size and isolation on the probability of a seed to germinate (*F*_1,160_ = 11.01, *p* = .001; *F*_1,160_ = 10.90, *p* = .001, respectively). The effect of population size was positive (Table 2), increasing from ~10% to ~20% probability of seed germination from small to large populations (Figure 4a). A similar effect size, but negative, was found for the isolation variable (Figure 4b; Table 2).

## 4 DISCUSSION

The mating system in *C. undulatum* is consistent with those found in the majority of proteaceous species (Collins & Rebelo, 1987). Total exclusion of insects from flowers resulted in our target species not being able to produce fruits, which demonstrated the requirement of visitation by a pollinator. The stigma, once triggered, flicks away from the anthers toward the lower tepals; this mechanism can only be activate once, and thus, the exclusion plus triggered flowers treatment showed that pollen grains exploded from the anthers were unable to reach the downward‐facing fertile part of the triggered style within the same flower, highlighting the efficacy of the trigger mechanism as a physical barrier to self‐pollination. This is similar to other observations on eastern Australian species in the genus, including *Conospermum taxifolium*, *Conospermum ericifolium*, *Conospermum ellipticum*, and *Conospermum longifolium* where no self‐pollination was found when pollinators were excluded (Morrison et al., 1994). Results from the hand self‐pollination treatment suggests that self‐incompatibility in *C. undulatum* was not only due to its specific flower morphology that prevents autogamy but was also a genetic response to prevent geitonogamy (i.e., self‐incompatibility).

We have demonstrated that habitat fragmentation, when combined with the *C. undulatum* mating system, has far reaching effects on the reproductive potential of the species. Against our initial expectations, fruit production responded to only one population descriptor, that being floral display, suggesting the only variable that affected the production of fruits was the capacity of a population to attract pollinators. This result shows the importance that floral display may have from a conservation point of view, particularly for mass‐flowering species that rely on huge floral displays to attract pollinators. Indeed, fragmentation of the habitat may result in patches that are not attractive for floral visitors due to a lack of resources. This result agrees with observations in other species where habitat fragmentation and its effect on floral display were the key determinant of pollinator abundance and, ultimately, fruit production (Delmas et al., 2014; Goulson, Lye, & Darvill, 2008). This is particularly important considering that the native bee *L. conospermi* (Hymenoptera) is likely to be the main pollen vector of *C. undulatum*, since hymenopterans are found to be more influenced by a reduction in floral display of mass‐flowering plants compared with dipterans (Delmas et al., 2014). Moreover, since a fruit can only develop after an insect visit, this suggests that the populations of *C. undulatum* with lower floral display index may be limited by pollen quantity due to a lack of pollinators. This may have a cascading effect on reproductive success and is worthy of further investigation, especially considering that pollen deposition and fruit production are essential steps in plant sexual reproduction.

A second, no less important step leading to seed production is the development of a healthy embryo. *Conospermum undulatum* is a resprouter plant, with a life‐history strategy adopted by 66%–80% of the plant species in the SWAFR (Bell, 2001). These species are able to regenerate vegetatively after disturbance, such as fire or herbivory, and their seed set is generally low (Lamont, Enright, & He, 2011). Nonetheless, although the seed production was expected to be low in *C. undulatum*, our results showed that the probability of setting seeds is doubled in large and connected populations with a high floral display index compared to small, isolated, and less attractive ones. The effect of habitat fragmentation on seed production has been investigated in numerous plant species with different compatibility systems and life‐history strategies and found to be detrimental (Aguilar et al., 2006; Aizen, Ashworth, & Galetto, 2002). In particular, these studies found that the reduction in the reproductive output was mainly due to disrupted interaction between plants and their pollinators following habitat fragmentation. The present study is consistent with these results and suggests that this negative effect can also be observed for mass‐flowering resprouter species. Moreover, if the lack of floral visitors was the only factor involved, it would have been reasonable to expect floral display to be the only significant factor for seed production, as it was for fruit production. However, in this case population size and isolation also became highly significant factors, consistent with our initial hypothesis. This suggests that besides the lack of floral visitors, genetic factors that prevent the development of the embryo and result in empty fruits may be present in small and isolated populations. Following our results of hand self‐pollination, it is reasonable to conclude that the recorded discrepancy between fruit production and seed production may reflect late acting self‐incompatibility, possibly due to an increased geitonogamy rate in small populations (Eckert, 2000), and/or inbreeding effects, resulting in a higher proportion of aborted seeds. Furthermore, the standardized regression coefficients demonstrate that population size was the most important variable in determining the production of seeds, with floral display and isolation also found to be important factors. These factors are essential considerations when planning conservation actions, such as translocations and reintroductions, in order to maintain adequate seed production in a population. In particular, the standardized ß coefficient of floral display was higher than that of isolation, underpinning the importance of floral display in the reproductive success of the mass‐flowering *C. undulatum*.

The last step in the (sexual) reproductive cycle is seed germination. We found patterns of response variable for germination to be similar to those of seed production, and in line with our initial hypothesis, namely that seeds produced in small and isolated populations resulted in a lower probability of germination. Since a viable seed has been produced, self‐incompatibility issues are drastically reduced at this point of the reproductive cycle of *C. undulatum*. Recent studies have found that in some cases increased isolation and a reduction in population size is not associated with an increase in biparental inbreeding (e.g., Byrne, Elliott, Yates, & Coates, 2007). This is due to an expansion of the usual foraging range of highly motile pollinators, such as birds or honeybees, in response to fragmentation. However, for plants pollinated by small, less‐motile pollen vectors, this is unlikely to be the case. This hypothesis was tested by Breed et al. (2015) in a case study of three *Eucalyptus* tree species; for the two small insect‐pollinated eucalypts, increased selfing and decreased pollen diversity were correlated with increased fragmentation, but no such relationship was evident for the bird‐pollinated eucalypt species. Therefore, our result is consistent with the hypothesized lack of extended gene flow able to rescue small and/or isolated populations from the effects of inbreeding (Aguilar, Quesada, Ashworth, Herrerias‐Diego, & Lobo, 2008; Honnay & Jacquemyn, 2007).

Although the Proteaceae are among the most widely studied Australian native plants, most research has focused on species of *Banksia*, *Hakea*, and *Grevillea*, with only a few studies specifically on *Conospermum* species. Moreover, most of the *Conospermum* research had different purposes being mainly focused on identifying the cues that stimulate seed germination (e.g., Tieu, Dixon, Meney, & Sivasithamparam, 2007), without taking into account other factors influencing seed production. Indeed, although germination of seeds is a crucial life‐history event, it may not inform conservation planning if considered on its own, because other important processes such as plant–pollinator interactions and gene flow are also likely to constrain reproduction.

This study has identified several aspects of the reproductive biology of *C. undulatum* that add to the growing base of knowledge of this genus and Proteaceae in general. Habitat fragmentation appears to be a significant threat to the future persistence of *C. undulatum*, and its effects were readily visible in the results of this study. Every stage of sexual reproduction was directly and significantly affected by aspects of habitat fragmentation. Ultimately, urban expansion on the Swan Coastal Plain may result in patches of native vegetation that are unattractive for pollinators, and too small and isolated to ensure long‐term population viability and adaptation ability based on reproduction by seeds. Future studies to help maximize the conservation effort should focus on clearly identifying the pollinator assemblage associated with successful pollination of this endemic species, as well as assessing the impact of habitat fragmentation on these essential floral visitors.

## CONFLICT OF INTEREST

The authors declare no conflicts of interest.

## AUTHOR CONTRIBUTIONS

ND led the writing of the manuscript, designed the experiment, collected, and analyzed the data; EJvE and WDS contributed to design the experiment and to data collection and analysis; MB helped improve the design of the experiment. All authors contributed critically to the drafts and gave final approval for publication.

## Data Availability

Data available from the Dryad Digital Repository https://doi.org/10.5061/dryad.4cg374r
