# Supplementary material for: Floral display and habitat fragmentation: Effects on the reproductive success of the threatened mass‐flowering Conospermum undulatum (Proteaceae)
Source: Ecol Evol. 2019 Sep 26;9(19):11494–503. doi: 10.1002/ece3.5653 (PMC6802041; doi:10.1002/ece3.5653)
Supplement: Supplementary file 1 [file ECE3-9-11494-s001.docx]

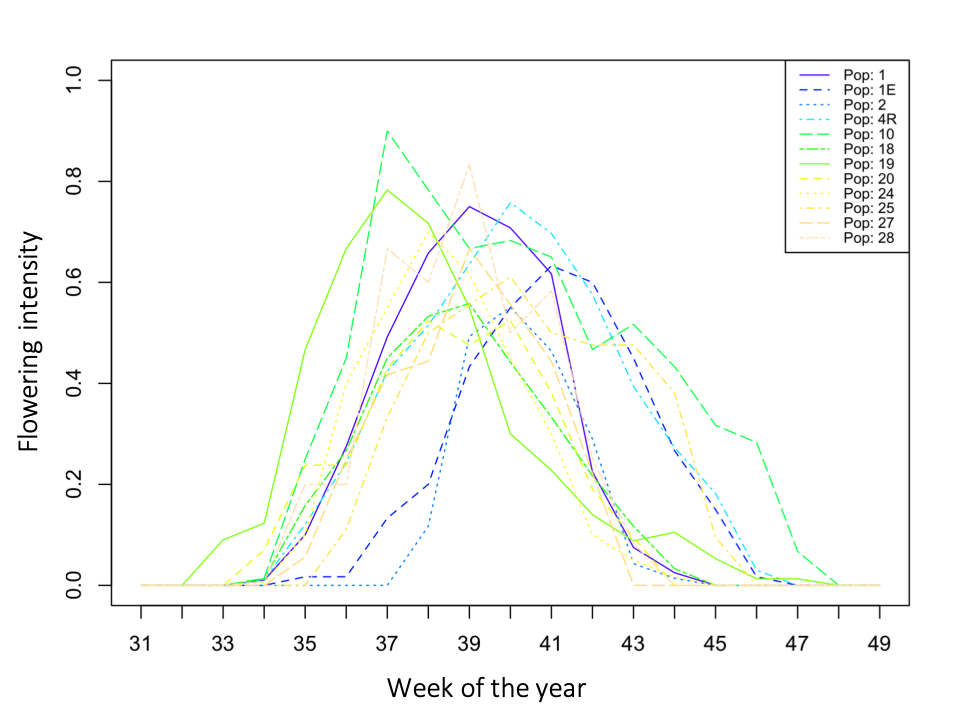


**Figure S1**. Flowering phenology of studied populations of C. undulatum across the entire flowering season.
