# Supplementary material for: Floral display and habitat fragmentation: Effects on the reproductive success of the threatened mass‐flowering Conospermum undulatum (Proteaceae)
Source: Ecol Evol. 2019 Sep 26;9(19):11494–503. doi: 10.1002/ece3.5653 (PMC6802041; doi:10.1002/ece3.5653)
Supplement: Supplementary file 2 [file ECE3-9-11494-s002.docx]

| **Predictor** | **Fruit production model** | **Seed production model** | **Seed germination model** |
| --- | --- | --- | --- |
| Population size | / | 0.240 (0.043) | 0.247 (0.074) |
| Isolation index | / | -0.156 (0.041) | -0.256 (0.078) |
| Floral display index | 0.314 (0.052) | 0.203 (0.050) | / |

**Table S1.** Standardised regression coefficient ß of predictors utilised in each model for variable comparison; standard error in parenthesis.
